# How telepresence and perceived enjoyment mediate the relationship between interaction quality and continuance intention: Evidence from China Zisha-ware Digital Museum

**DOI:** 10.1371/journal.pone.0317784

**Published:** 2025-01-22

**Authors:** Mengjie Shi, Liyuan Deng, Mingshan Zhang, Yanghuan Long

**Affiliations:** 1 School of Design, Jiangnan University, Wuxi, China; 2 College of Art & Design, Nanjing Forestry University, Nanjing, China; University of Leeds, UNITED KINGDOM OF GREAT BRITAIN AND NORTHERN IRELAND

## Abstract

The rapid development of digital technologies and the diversification of user needs have made digital museums significant platforms for cultural dissemination and education, attracting unprecedented attention. This study aims to investigate how interaction quality influences users’ psychological responses and their continuance intention to use digital museums. By integrating the Stimulus-Organism-Response (S-O-R) model and telepresence theory, this study proposes a novel model to examine users’ continuance intention. A questionnaire survey of visitors to the China Zisha-ware Digital Museum (CZDM) yielded 414 valid responses, which were analyzed using Partial Least Squares Structural Equation Modeling (PLS-SEM). The results indicate that: 1) The interaction quality of digital museums (i.e., interactivity, vividness, and authenticity) and users’ psychological responses (i.e., telepresence, perceived enjoyment) significantly affect users’ continuance intention to varying degrees. 2) Telepresence significantly mediates the relationship between interaction quality and continuance intention. 3) Perceived enjoyment serves as an important mediator between interactivity, vividness, and continuance intention, while its mediating role between authenticity and continuance intention is not significant. This study offers theoretical and practical insights for optimizing digital museum user experience design to enhance continuance intention, while also promoting the dissemination of digital culture and supporting educational efforts.

## 1. Introduction

As crucial cultural institutions and tourist attractions, museums have been experiencing rapid growth in recent years [1,2]. As data shows, the number of museums worldwide has increased from 22,000 [3] in 1975 to 104,000 [4] today. The number of museums in China has increased from 3,866 in 2021 to 6,833 in 2023, with an average of one new museum added every 1.2 days. This has attracted a record high of 1.29 billion visitors [5]. However, despite the growing number of museums and visitors, these institutions face challenges such as intense market competition and diverse user demands. In response, museums are increasingly exploring digital solutions [6]. The Louvre Museum [7], the Palace Museum [8], and the Smithsonian National Museum of Natural History [9] are notable examples of museums that have adopted digital strategies. Statistics from the National Cultural Heritage Administration [10] indicate that the number of online exhibitions launched by museums across China increased from over 3,000 in 2021 to nearly 10,000 in 2022. This resulted in online viewership approaching 1 billion, and new media viewership exceeding 10 billion [11]. It can be seen that the digital museum boom has emerged.

Digital museums employ technologies such as augmented reality (AR), virtual reality (VR), and mixed reality (MR) to create online virtual showrooms via official websites and mobile applications. With the advent of these technologies, museums have gradually shifted from an “object-centric” paradigm to an “experience-centric” one [12,13]. Compared to traditional museums, digital museums offer more flexible access methods, richer interactive experiences, and personalized guide services. Firstly, digital museums utilize visual images to create a rapid communication channel between virtual information and the real world, overcoming the time and space limitations of traditional museums. In traditional museum settings, collections are displayed using cabinets, shelves, and physical exhibits [14]. However, digital museums utilize high-definition images, videos, and virtual reality technologies to create virtual exhibition scenes. For example, combining real-world images and videos with 360° VR enables users to experience an immersive panoramic environment [15]. Such experiences are accessible anytime, anywhere, and across various devices including mobile phones, tablets, and computers. This obviates the need for specialized VR apparatus, such as helmets or eyewear [16]. Secondly, digital museums integrate a variety of multimedia resources, including videos, audio, and animations, which enable more detailed exhibits, vivid visual presentations, and interactive feedback. This enhances user engagement by providing immersive experiences and improving their access to, communication with, and understanding of digital museum collections, thereby strengthening the connection between digital museums and their users [17,18]. Thirdly, digital museums transcend the constraints of traditional fixed display forms, encouraging users to explore non-linearly and choose content based on their interests and needs. Consequently, the user experience has shifted from passive acceptance to active exploration [19].

Given the evolving museum experience, studying users’ continuance intentions is crucial for optimizing digital museum services. Continuance intention aligns seamlessly with the core objective of digital museums, namely, to cultivate sustained user engagement [20]. Current research on digital museum users’ continuance intention mainly focuses on three aspects: 1) The impact of new digital technologies. These studies demonstrate how new technologies, such as AR and VR, significantly enhance users’ willingness to engage with museums. They not only boost users’ interest in museum engagement [16] but also encourage them to share their experiences [21]. 2) Evaluation of user behavioral characteristics, including search patterns [22], evaluation attributes [23], and requirements [24]. These studies offer critical data for understanding user behavior and needs, which can inform the development of targeted strategies to enhance continuance intention. 3) Evaluation of influencing factors. These evaluations first consider design factors in digital museums, such as interface design [25], aesthetic presentation [26], interaction quality [19], and information quality [21]. Secondly, they investigate user response factors, including satisfaction [27], self-cognition [28], arousal [24], and confirmation [29].

Existing research has made significant contributions to our understanding of the continuance intention of digital museum users. However, it has predominantly focused on design factors and the direct effects of user responses. There is a paucity of comprehensive research on how the unique properties of digital museums (e.g., interactivity, vividness, authenticity) stimulate users’ psychological responses and drive continuous engagement. In particular, the psychological states, such as telepresence and enjoyment, generated during user-system interactions are considered crucial to user experience quality [30,31]. Therefore, it is essential to deeply explore the relationship between users’ psychological reactions and continuance intention.

The stimulus-organism-response (S-O-R) theory offers a framework for understanding how external environmental factors (stimuli) influence an individual’s internal states (organisms), leading to specific observable behaviors (responses) [32]. Scholars have applied the S-O-R theory to various fields, including online shopping [33], social commerce [34], smart services [35], and tourism [36]. The S-O-R theory is equally applicable to virtual experiences, showing how technical features impact users’ perceptions and behavioral intentions [37,38]. In this study, “stimuli” refers to the virtual elements of digital museums, including interactivity, vividness, and authenticity. “Organism” represents users’ mental experiences, such as telepresence and perceived enjoyment. “Response” describes users’ attitudes and willingness to engage with the digital museum. Digital technologies, such as augmented reality, which enhance interactivity, immersion, and realism [39], significantly shape users’ perceptions [40] and sensory experiences [41]. Consequently, this study focuses on digital technology attributes related to user experience to explore the core factors influencing continuance intentions towards digital museum platforms.

The value of this research lies in three key innovations. First, it extends the S-O-R and telepresence theories by applying them to the emerging field of digital museums. Second, it proposes a new user experience model that links interaction quality with psychological factors, exploring both the direct and indirect mechanisms influencing users’ continuance intention. This model provides a theoretical foundation for designing user-centric experiences in digital museums. Finally, the findings offer practical insights for practitioners to harness digital technologies, improve user experience design and promote sustainable growth. Collectively, these efforts advance the innovation and growth of digital cultural services.

The following section outlines the research structure. Section 1 introduces the topic; Section 2 develops the research hypotheses and model; Section 3 details the measurement processes and data collection methods; Section 4 presents the analysis results; Section 5 discusses the results of the hypothesis testing; finally, Section 6 highlights the implications, limitations, and directions for future research.

## 2. Research hypotheses and model development

### 2.1 User perception of digital museum service as stimuli (S)

#### 2.1.1 Interactivity

“Interactivity” describes the capacity of technology to facilitate user interaction and participation with content [42]. In virtual environments, effective interactions enhance users’ positive emotions [43], making user-virtual museum interactions a stimulus-driven variable. Interactivity affects users’ ability to create, alter, and control media content [44], thereby allowing them to actively engage with or manipulate information based on personal preferences [45]. Interactivity typically encompasses three aspects: speed, scope, and mapping of controls to the virtual world [46]. The speed of media response when users interact with content is a key factor in determining the level of interactivity. Scope refers to the extent users can manipulate content within an interactive environment. Mapping describes the degree of correspondence between the controls in the virtual environment and those in the real world. In virtual environments, these interactive components coexist, enabling users to zoom in or out, view collections from various angles and distances, browse information, and interact with online services. Nowadays, advancements in technology have significantly enhanced the interactivity of online platforms by facilitating the presentation of virtual objects in a more natural and realistic manner [47], effectively bridging the gap between physical objects and users’ imaginations. Research indicates that media interaction can positively influence decision-making, with higher levels of interaction leading to greater user satisfaction [48].

#### 2.1.2 Vividness

Vividness, also referred to as realness or richness [49], signifies “the representational richness of a mediated environment” [46] or “vibrant sensory stimulation” [50]. Vividness is measured in two main dimensions: depth and breadth. Depth pertains to the quality of information perceived by users, including color and image fidelity. Breadth involves the range of sensory dimensions (such as color, audio, and animation) provided by the interactive environment [51]. For instance, color images are more vivid than text, and animations are more vivid than still images [52]. Studies have demonstrated that highly vivid displays can create strong real-world impressions for users and stimulate cognitive processing [53], enhancing their psychological perception of future consumption or service experiences [39]. Compared to traditional museums, digital museums utilize technology to engage multiple human senses [47], offering users a higher level of vividness. They employ bright colors, lifelike images, and pleasant sounds to provide strong visual and auditory stimulation. Furthermore, digital museums can convey a sense of touch through the sounds of objects colliding in the virtual world [54]. These multisensory feedback mechanisms enhance users’ perception of depth and breadth, ultimately stimulating higher levels of positive emotions [55]. Therefore, this study considers vividness as a crucial stimulus variable in the context of digital museums.

#### 2.1.3 Authenticity

Authenticity, a pivotal concept in cultural tourism studies, typically describes the degree of originality and integrity of objects (such as cultural relics or events) in the material world [56], and is extensively applied in museum studies, cultural activities, and heritage sites [57,58]. Despite the lack of consensus among academics on the definition of authenticity [59], Wang categorizes it into three types from the perspective of tourism experiences: objective authenticity, constructive authenticity, and existential authenticity [60]. Objective authenticity refers to the accuracy or genuineness of the tourist object, including related places, processes, activities, or cultural relics [61]. Constructive authenticity posits that tourists’ subjective evaluation of a scene or experience is central. When tourists perceive an object as real, it is due to symbolic authenticity rather than the object’s inherent authenticity [62]. In other words, tourists will consider a replica “real” if it accurately reflects the key characteristics of the tourism object [63]. Consequently, constructive authenticity is contingent upon the tourists’ personal expectations and perceptions. Conversely, existential authenticity is less concerned with the authenticity of the tourism object [60] and more focused on how tourists experience their own identities or perceive their own existence in specific tourist settings [64]. In virtual environments, users cannot physically interact with objects and must rely on images, sound, video, and other media to understand them. Research indicates that users associate this information with their prior knowledge or expectations to form a composite image, which they then compare with the target object to evaluate its authenticity (e.g., similarity, completeness, and accuracy) [41]. For researchers and practitioners, understanding whether users perceive the museum experience as authentic and meeting their expectations is crucial. Therefore, this study adopts the concept of constructive authenticity, viewing it as a process where users construct their own understanding of authenticity based on their prior knowledge and expectations [65,66].

### 2.2 Internal psychological response as an organism (O)

#### 2.2.1 Telepresence

Telepresence is a key concept for understanding users’ mental states and behavior in virtual experiences [39]. It is defined as the degree to which a person feels present in a mediated environment rather than in the immediate physical environment [46]. It is not about direct sensory input but about using technology to create the sensation of being in another place [67]. Telepresence is widely used to explain user experiences in various contexts, including virtual reality shopping [68], mobile learning systems [69], and virtual travel [70]. For example, Jung-Hwan Kim et al. utilized telepresence theory to examine users’ authentic sentiments and consumption behavior in a virtual reality furniture store [39]. A primary goal of digital museums is to immerse users in virtual scenes using technologies such as AR, allowing them to personally experience history and art. Therefore, this study uses telepresence theory to explore the psychological mechanisms and behavior of users in digital museums to better understand their perceptions and attitudes.

Numerous studies show that interactivity and vividness are crucial for enhancing users’ telepresence experiences in virtual environments [45,71]. Interactivity allows users to achieve a sense of telepresence by manipulating and controlling objects in real time. For example, in a virtual museum, users can explore and interact with all elements by overturning, rotating, enlarging and reducing, and moving forward or backward, thereby developing a profound sense of engagement and immersion. Such immersive experiences transcend the limitations of physical space and lead users into a deep state of telepresence. Vividness, characterized by multiple sensory channels and high-quality information presentation, enhances users’ sensory and cognitive arousal, increasing their immersion [72,73]. Furthermore, studies emphasize the importance of authenticity in enhancing telepresence [43,74]. This creates the sensation in users that they are in a real museum, able to approach authentic exhibits and receive assistance, thereby attaining a sense of presence. Thus, we hypothesized:

Interactivity (**H1a**), vividness (**H2a**), and authenticity (**H3a**) have a positive impact on telepresence.

#### 2.2.2 Perceived enjoyment

Perceived enjoyment measures the pleasure and excitement users experience when using a system [75]. Kim et al. defined enjoyment in VR as consumers’ affective responses, including feelings of pleasure, fun, and happiness [38]. In this study, perceived enjoyment refers to the pleasure users experience when using digital museums. Advances in virtual technology that overcome the limitations of time and space have increased users’ demand for perceived enjoyment, such as visual and interactive pleasure [76,77].

In virtual experiences, there is a positive correlation between interactivity and vividness and users’ perceived enjoyment. First, a highly interactive virtual world provides users with autonomy and control [78], which increases their perceived enjoyment of virtual objects [79]. Conversely, slow system responses can lead to user impatience and abandonment [80,81]. Second, higher vividness typically offers more sensory cues and sensory channels, making the experience more emotionally engaging [82]. For example, Jung-Hwan Kim et al. and Yim et al. found that vivid displays, including clear images and vibrant colors, rich sensations, and easy accessibility increase user enjoyment [39,42]. Additionally, Kichan Nam et al. found that authenticity is positively related to enjoyment in VR travel experiences [83]. In summary, authentic presentation brings users’ online experiences closer to reality, evoking pleasure and excitement. Thus, we hypothesized:

Interactivity (**H1b**), vividness (**H2b**), and authenticity (**H3b**) have a positive impact on perceived enjoyment.

### 2.3 Continuance intention as a response (R)

#### 2.3.1 Telepresence, perceived enjoyment and continuance intention

Continuance intention refers to users’ willingness to continue using a product or service in the future based on their past experiences [19]. It is a key indicator of a product or service’s success or failure [20]. Once a product has lost its initial novelty, there is an increased likelihood of its success if users’ continuance intention for the product or information system increases [84]. In this study, continuance intention is defined as the extent to which users anticipate using digital museums in the future. It is significantly influenced by both positive and negative emotions. In virtual environments, telepresence, a crucial psychological experience, positively influences users’ decision-making and behavior [85]. In other words, higher levels of telepresence lead users to favor virtual reality applications, thereby further facilitating their continuance intention [86].

Similarly, perceived enjoyment as an emotional state also influences users’ intentions to continue using the technology [68,87]. Interacting with technologies that are perceived as interesting and enjoyable creates an expectation of psychological reward in users, which in turn encourages further engagement [88]. Numerous studies confirm that perceived enjoyment significantly impacts continuance intentions in various contexts, including online shopping, online gaming, and mobile travel applications [89,90]. However, research on digital museums is limited. Users tend to regard virtual experiences as more personalized, attractive, and interesting than traditional display interfaces [91]. Consequently, it can be hypothesized that digital museums using virtual technology significantly enhance user enjoyment, thereby increasing their willingness to continue using them. Thus, we hypothesized:

Telepresence (**H4a**) and perceived enjoyment (**H4b**) have a positive impact on continuance intention.

#### 2.3.2 Interactivity, vividness, authenticity and continuance intention

Researchers have demonstrated the significant impact of interactivity on continuance intention. Mandari et al. indicated that in e-government systems, high interactivity leads users to continue using the system for information and services [92]. Ye et al. and Khare et al. found similar results in studies of online travel sites, showing that site interactivity is related to users’ intentions to return [93,94]. Additionally, Xiaolu Hu, in research on VR systems for intangible cultural heritage, noted that interactivity refers to communication between the user and the system [95]. Interaction with VR systems allows users to navigate and participate actively, leading to more frequent and sustained use.

Recent studies have highlighted the significance of vividness in shaping users’ continuance intention in virtual experiences. Foroughi et al. found that in virtual tourism, vividness is a key factor promoting positive emotional experiences, which subsequently influences users’ willingness to continue using virtual experiences [24]. Similarly, W. Kim et al. found that higher levels of vividness can increase the attractiveness of information and systems, thereby enhancing their appeal to users in e-commerce contexts [96]. Consequently, scholars recommend that the vividness of virtual technologies, such as AR, should be a key indicator for predicting users’ continuance intention [95].

Authenticity also significantly impacts users’ continuance intention toward virtual experiences. Research indicates that realistic experiences provided by technologies such as mobile information technology and VR positively influence users’ continuance intention and engagement in virtual experiences [97]. For instance, in the context of virtual tourism of cultural heritage sites, if the virtual technology offers a sufficiently authentic experience, users may view it as a substitute for traditional travel [98]. Consequently, authenticity influences users’ behavioral intentions. The greater the perceived authenticity, the greater the users’ willingness to engage in virtual experiences [97]. Thus, we hypothesized:

Interactivity (**H5a**), vividness (**H5b**), and authenticity (**H5c**) have a positive impact on continuance intention.

#### 2.3.3 The mediating role of telepresence and perceived enjoyment

The analysis showed that, in virtual experiences, interactivity, vividness, and authenticity significantly increase users’ telepresence and perceived enjoyment. These factors further enhance users’ continuance intention. According to Hayes [99], a mediator variable is the mechanism through which the independent variable affects the dependent variable. Therefore, we speculate that telepresence and perceived enjoyment each play mediating roles in this process. Thus, we hypothesized:

Telepresence mediates the relationship between interactivity (**H6a**), vividness (**H6b**), authenticity (**H6c**) and continuance intention.

Perceived enjoyment mediates the relationship between interactivity (**H7a**), vividness (**H7b**), authenticity (**H7c**) and continuance intention.

**Fig 1:**
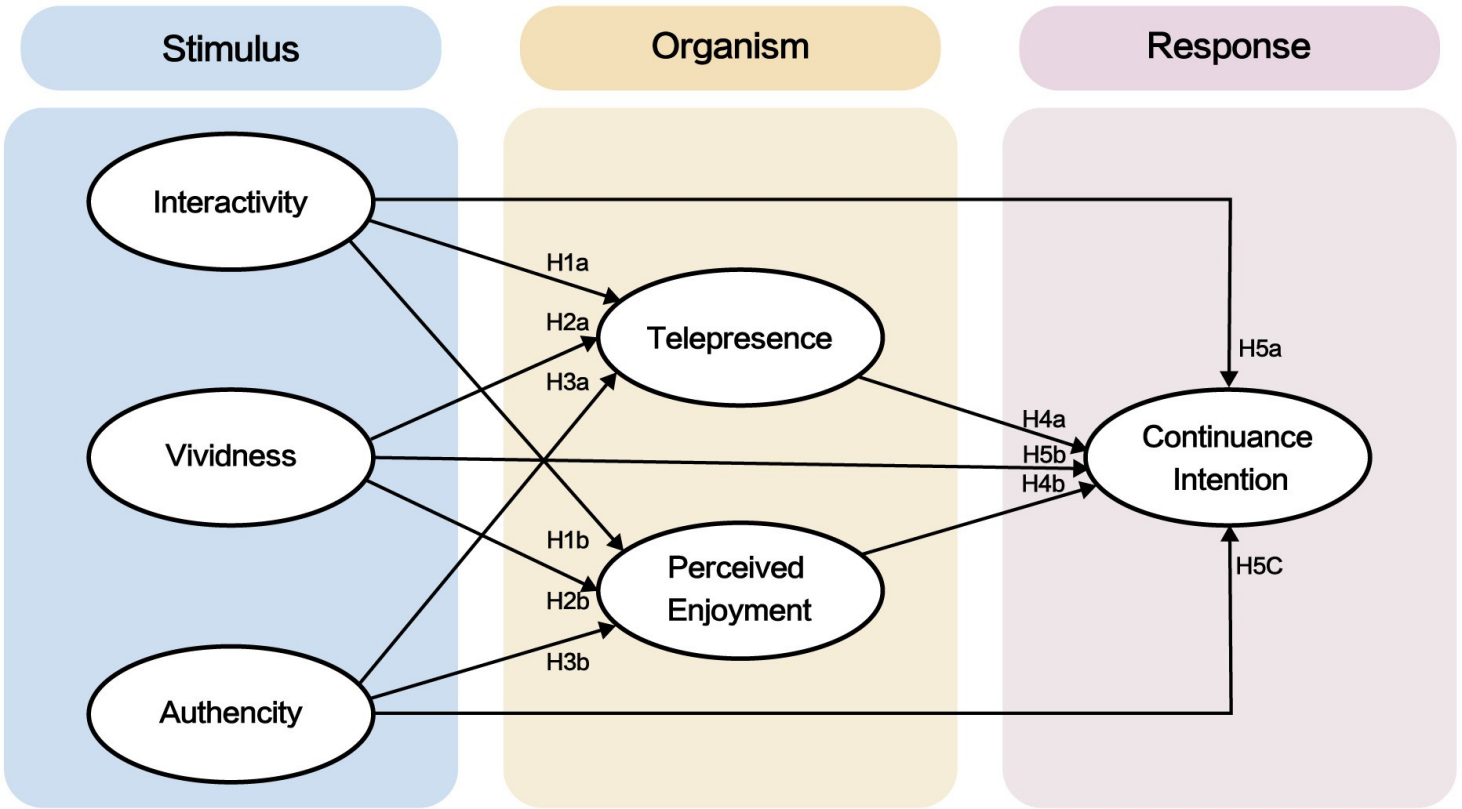
Research model.

Based on the 17 hypotheses, this study proposes the following models (Fig 1).

## 3. Research methodology

Partial Least Squares Structural Equation Modeling (PLS-SEM) was selected for data analysis due to its ability to optimize model fit by minimizing squared residuals between observed and predicted values, making it particularly suitable for handling non-normal data and complex models [100]. PLS-SEM employs the bootstrap method to evaluate the confidence intervals of path coefficients, which enhances the robustness of the results. Widely used in fields such as management, information systems, and market research [101,102], PLS-SEM is well-suited for analyzing the interaction effects in this study.

### 3.1 Stimulus website

**Fig 2:**
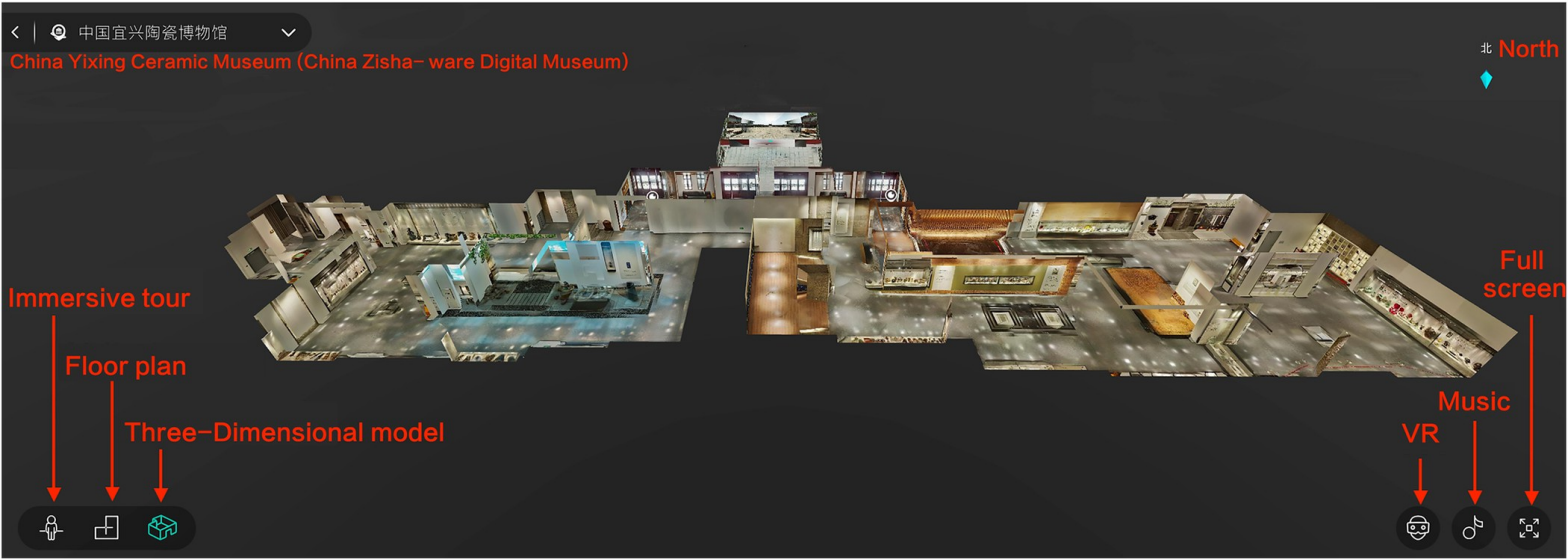
Three-Dimensional model of the China Zisha-ware digital museum.

**Fig 3:**
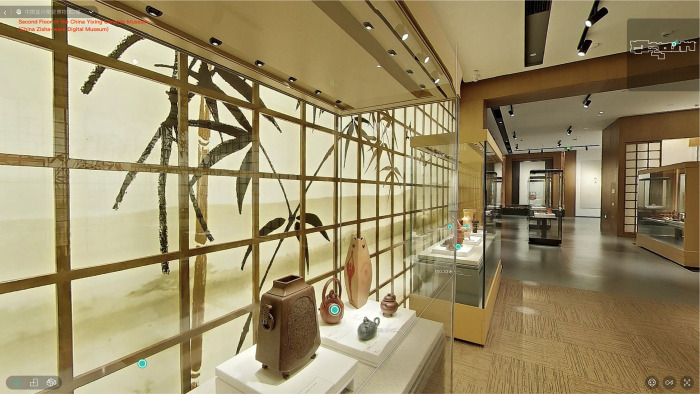
Virtual exhibition hall of China Zisha-ware digital museum.

This study employs the China Zisha-ware Digital Museum (CZDM), a free online resource, as its investigation site [103] (Figs 2 and 3). Launched online in May 2022, the CZDM primarily exhibits the Yixing zisha pottery, which is one of China’s most renowned pottery types [104]. The museum integrates advanced digital technologies, including panoramic digital technology and VR, allowing users to overcome time and space constraints and immerse themselves in the culture of zisha pottery [105]. The museum also offers high-quality visual presentation, a user-friendly interface, vibrant color schemes, and a clear navigation system, serving as a model for local and small-to-medium-sized digital museums. Thus, the CZDM serves as an ideal case study for this research.

### 3.2 Questionnaire design

A questionnaire survey was conducted to verify the 17 hypotheses proposed in the previous section. The questionnaire consisted of two parts: the first part collected demographic information (gender, age, education level, and occupation, etc.) to assess the sample’s representativeness. The second part measured the research variables using a seven-point Likert scale (1 = “strongly disagree”; 7 = “strongly agree”) to ensure reliability and validity [106]. After drafting the initial questionnaire, a pre-test with 50 participants was conducted, and two experts in related fields were consulted. Based on the feedback, question clarity was improved and language refined. Finally, the revised questionnaire included six variables and 22 measurement items. Further details can be found in Table 1.

**Table 1:** Measurement model and sources.

Construct	Indicator	Description	References
Interactivity (INT)	INT1	In the CZDM, I can control my movements.	Yim et al. [42]
	INT2	In the CZDM, I can control my position.	
	INT3	In the CZDM, I can choose the exhibits I want to see.	
	INT4	In the CZDM, my needs are promptly and effectively addressed.	
Vividness (VIV)	VIV1	The images in the CZDM are very clear.	Yim et al. [42]; Wen-Chin Hsu et al. [43]
	VIV2	The exhibits displayed in the CZDM are very realistic.	
	VIV3	The CZDM provides exhibit information that stimulates multiple senses (e.g., images, sound, and VR).	
Authenticity (AUT)	AUT1	The CZDM looks real.	Nam et al. [83]
	AUT2	The CZDM is as accurate as the real attractions.	
	AUT3	The CZDM accurately reproduces real objects virtually.	
	AUT4	In the CZDM, I can discern the real features of the attractions.	
Telepresence (TE)	TE1	In the CZDM, I feel immersed in its virtual world.	Hyeon-Cheol Kim [107]; Nathalie Spielmann [73]
	TE2	In the CZDM, my body is in the real world, but my mind is in the virtual one.	
	TE3	In the CZDM, I forget that I’m in an experiment.	
	TE4	The world generated by the CZDM feels like “somewhere I visited” rather than “something I saw”.	
Perceived Enjoyment (PE)	PE1	Using the CZDM is very interesting.	Jung-Hwan Kim, et al. [39]; Moon and Kim [87]
	PE2	Using the CZDM is very enjoyable.	
	PE3	Using the CZDM does not make me feel bored.	
	PE4	The CZDM has sparked my curiosity to continue using it.	
Continuance Intention (CI)	CI1	I intend to continue using the virtual museum.	Bhattacherjee [84]
	CI2	I intend to use the virtual museum more in the future.	
	CI3	If possible, I would recommend the virtual museum to my friends.	

### 3.3 Sample and data collection

Ethical approval for this study was granted by the Jiangnan University Ethics Committee (JNU202406RB026). All procedures conformed to the ethical standards of the institutional research committee, the 1964 Declaration of Helsinki, and its subsequent amendments or comparable ethical standards. Electronic written informed consent was obtained from all participants before they began the questionnaire. This study did not involve any minors.

The questionnaire was distributed via Credamo, a widely used online survey platform in China, between May and October 2024. Prior to the survey, participants were informed about the study’s nature and purpose, emphasizing complete anonymity and that no identifying information would be collected. They were also assured of their right to withdraw at any time by simply closing the survey window without consequences. However, as the questionnaire was completely anonymous, the submitted responses could not be traced or removed.

We included an introduction to the CZDM’s usage and main functions, using pictures and text to ensure that participants were familiar with the website. To enhance data reliability, a preliminary multiple-choice question confirmed completion of the CZDM tour, without which participants could not proceed. We also included a test question with a correct answer; unmatched responses were deemed invalid. Finally, Credamo’s IP restriction feature was activated to allow only one response per IP address, preventing duplicate responses. A total of 438 questionnaires were received; 414 were valid (94.52% acceptance rate). Exclusions included responses under 90 seconds or clearly insincere answers [19,108]. The sample size of valid responses exceeds 10 times the number of items [22], satisfying the sample requirements for SEM [109].

### 3.4 Descriptive statistics

The SPSS 27.0 software was used for descriptive statistical analysis, as detailed in Table 2. Among the respondents, 54.6% were male and 45.4% were female. In terms of educational attainment, the largest group held bachelor’s degrees (54.6%), followed by those with junior college degrees or below (19.6%). Most respondents (63.8%) were between the ages of 18 and 30, which is consistent with young people’s preference for digital technology [28].

**Table 2:** Demographic information of respondents.

Sample	Categories	Number	Percentage
**Gender**	Male	226	54.6
	Female	188	45.4
**Age(years)**	18–30	264	63.8
	31–40	66	15.9
	41–50	59	14.3
	>50	25	6
**Education**	Junior college degree or below	81	19.6
	Bachelor degree	226	54.6
	Master’s degree	72	17.4
	Doctoral degree	35	8.4
**Occupation**	Student	222	53.6
	Researcher	43	10.4
	Cultural enthusiast	85	20.5
	Others	64	15.5

To comprehensively evaluate data normality, skewness, and kurtosis, the Kolmogorov-Smirnov test, the Shapiro-Wilk test, and the Jarque-Bera test were performed. Skewness indicates the symmetry of a data distribution, while kurtosis reflects its peakedness. According to Kline [110] and Leech et al. [111], absolute skewness values below 3 and kurtosis values below 10 are generally considered acceptable. Our results showed that skewness ranged from -0.259 to 0.021, and kurtosis from -0.883 to -0.486, both within acceptable limits, indicating that the data distribution is approximately symmetric and flat, closely aligning with normality.

However, the Kolmogorov-Smirnov and Shapiro-Wilk tests produced p-values <0.001 for all variables, indicating significant deviations from normality. Similarly, the Jarque-Bera test produced p-values <0.001 for all variables, further confirming these deviations. This may be attributed to the large sample size (n = 414), which makes normality tests overly sensitive to minor deviations. To address this, we employed PLS-SEM for analysis, as it provides robust model estimation under conditions of non-normal data distribution [100].

### 3.5 Correlation analysis

Pearson correlation analysis was conducted to preliminarily assess the linear relationships among the observed variables. The results indicated that most variables were significantly positively correlated (p < 0.01), providing empirical support for the subsequent SEM model analysis. Moreover, the correlation coefficients ranged from 0.247 to 0.405, remaining well below the multicollinearity threshold of 0.8 [112], which indicates a low likelihood of multicollinearity issues in the model.

### 3.6 Variance inflation factor (VIF) test

The VIF is used to detect multicollinearity among variables. In this study, all VIF values range between 1.738 and 2.315, which are below the commonly cited thresholds of 10 [113], 3.3 [114], and 3 [115]. The results suggest that multicollinearity is not a concern among the independent variables, ensuring model estimation stability.

## 4. Data analysis and model validation

SmartPLS 4.0 software was used for PLS-SEM analysis. Data analysis was conducted in three stages: 1) Common Method Bias and Endogeneity Tests: This stage addresses potential spurious covariance and measurement errors arising from identical measurement tools or methods. The Endogeneity Test assesses whether unobserved confounders or endogenous relationships bias model estimates, ensuring structural model validity. 2) Measurement Model: This stage evaluates the validity and reliability of constructs within the hypothesized model. 3) Structural Model: This stage employs path analysis to test research hypotheses and evaluate the direct, indirect, and total effects of independent on dependent variables.

### 4.1 Common method bias test and endogeneity test

Since the data originate from a single source, common method bias (CMB) may influence the relationships between variables, potentially leading to inaccurate model estimates [116]. Therefore, this study employs Harman’s single-factor test, following Podsakoff et al. [117]. An unrotated principal component analysis was conducted on 22 items, resulting in the extraction of six factors with eigenvalues exceeding 1. The variance explained by factor 1 was 31.6%, below the 50% threshold, indicating no presence of CMB in this study [118].

The Gaussian copula method was employed in SmartPLS to assess the potential endogeneity issue [119]. This instrument-free statistical approach is effective in situations where identifying valid instruments is challenging. Additionally, it addresses endogeneity by modeling the joint distribution of the endogenous variable and the error term, allowing inferences on model parameters through maximum likelihood estimation. The p-values for all tested relationships between endogenous variables exceeded 0.05 (Table 3), indicating no significant endogeneity concerns.

**Table 3:** Results of the Gaussian copula test for endogeneity assessment.

Path	Original sample (O)	Sample mean (M)	Standard deviation (STDEV)	T statistics (|O/STDEV|)	P values
GC (AUT) → TE	0.146	0.102	0.255	0.573	0.567
GC (AUT) → PE	-0.214	-0.281	0.343	0.624	0.533
GC (AUT) → CI	0.516	0.432	0.279	1.848	0.065
GC (INT) → TE	-0.053	-0.045	0.213	0.248	0.804
GC (INT) → PE	0.334	0.318	0.264	1.266	0.206
GC (INT) → CI	-0.333	-0.258	0.245	1.362	0.173
GC (VIV) → TE	0.095	0.050	0.207	0.456	0.648
GC (VIV) → PE	0.245	0.183	0.213	1.152	0.249
GC (VIV) → CI	0.277	0.197	0.226	1.224	0221
GC (TE) → CI	-0.349	-0.258	0.309	1.130	0.259
GC (PE) → CI	0.061	0.079	0.277	0.222	0.825

*Note*. →represents the path relationship.

### 4.2 Measurement model evaluation

Cronbach’s α and composite reliability (CR) were used to assess internal consistency, while convergent validity was evaluated through average variance extracted (AVE) and item loading. Cronbach’s α, CR values above 0.7, and AVE values over 0.5 with factor loadings exceeding 0.7 indicate good internal consistency and effective convergence on latent variables [120,121]. Table 4 shows that all reliability and validity metrics meet these thresholds.

**Table 4:** Reliability and validity test results.

Construct	Item	Standardized factor loading	Cronbach’s α	CR	AVE
INT	INT1	0.839	0.880	0.881	0.736
	INT2	0.866			
	INT3	0.865			
	INT4	0.862			
VIV	VIV1	0.876	0.841	0.848	0.758
	VIV2	0.885			
	VIV3	0.850			
AUT	AUT1	0.823	0.844	0.847	0.680
	AUT2	0.825			
	AUT3	0.822			
	AUT4	0.829			
TE	TE1	0.848	0.870	0.870	0.719
	TE2	0.840			
	TE3	0.853			
	TE4	0.850			
PE	PE1	0.841	0.856	0.858	0.698
	PE2	0.838			
	PE3	0.856			
	PE4	0.807			
CI	CI1	0.857	0.815	0.817	0.729
	CI3	0.857			
	CI4	0.848			

Discriminant validity ensures low correlations and significant distinctions among latent variables. When a variable’s correlation with others is lower than the square root of its AVE, discriminant validity is confirmed. In Table 5, bolded values represent the square root of each variable’s AVE, all of which exceed inter-variable correlations, indicating strong discriminant validity. The Heterotrait-Monotrait (HTMT) ratio further confirmed discriminant validity [122]. Table 6 shows that HTMT values for all latent variables are below 0.9 [123], further supporting the model’s robust discriminant validity.

**Table 5:** Discriminant validity test results (Fornell and Larcher criterion).

	TE	PE	CI	INT	VIV	AUT
**TE**	**0.848**					
**PE**	0.388	**0.836**				
**CI**	0.455	0.441	**0.854**			
**INT**	0.517	0.417	0.468	**0.858**		
**VIV**	0.486	0.442	0.417	0.487	**0.871**	
**AUT**	0.421	0.313	0.366	0.448	0.357	**0.825**

*Note*. The items on the diagonal on bold represent the square roots of the AVE.

**Table 6:** Discriminant validity test results (HTMT ratio).

	TE	PE	CI	INT	VIV	AUT
**TE**						
**PE**	0.450					
**CI**	0.539	0.527				
**INT**	0.590	0.479	0.551			
**VIV**	0.565	0.517	0.501	0.565		
**AUT**	0.490	0.365	0.438	0.517	0.421	

### 4.3 Structural model assessment

This study first evaluated the overall model fit using the standardized root mean square residual (SRMR) and normed fit index (NFI). The SRMR value was 0.046, which is below the recommended threshold of 0.08, indicating a good model fit [124]. The NFI value was 0.866, which is close to 1, suggesting an acceptable model fit.

Next, the explanatory power of each endogenous variable was assessed using the coefficient of determination (R^2^) [100]. In this study, R^2^ values ranged from 25.8% to 36.7%, above the acceptable threshold of 19% [125], indicating reasonable explanatory power for each construct (Table 7). To further validate the model’s predictive capacity, this study employed the predictive relevance (Q^2^) [100]. Q^2^ values of 0.259, 0.175, and 0.239 were all above zero, indicating acceptable predictive relevance [126] (Table 7). Q^2^ values of 0.02, 0.15, and 0.35 represent small, medium, and large predictive relevance, further supporting the model’s predictive capability.

**Table 7:** Results of R^2^ and Q^2^.

Constructs	R^2^	Q^2^
**TE**	0.367	0.259
**PE**	0.258	0.175
**CI**	0.346	0.239

**Fig 4:**
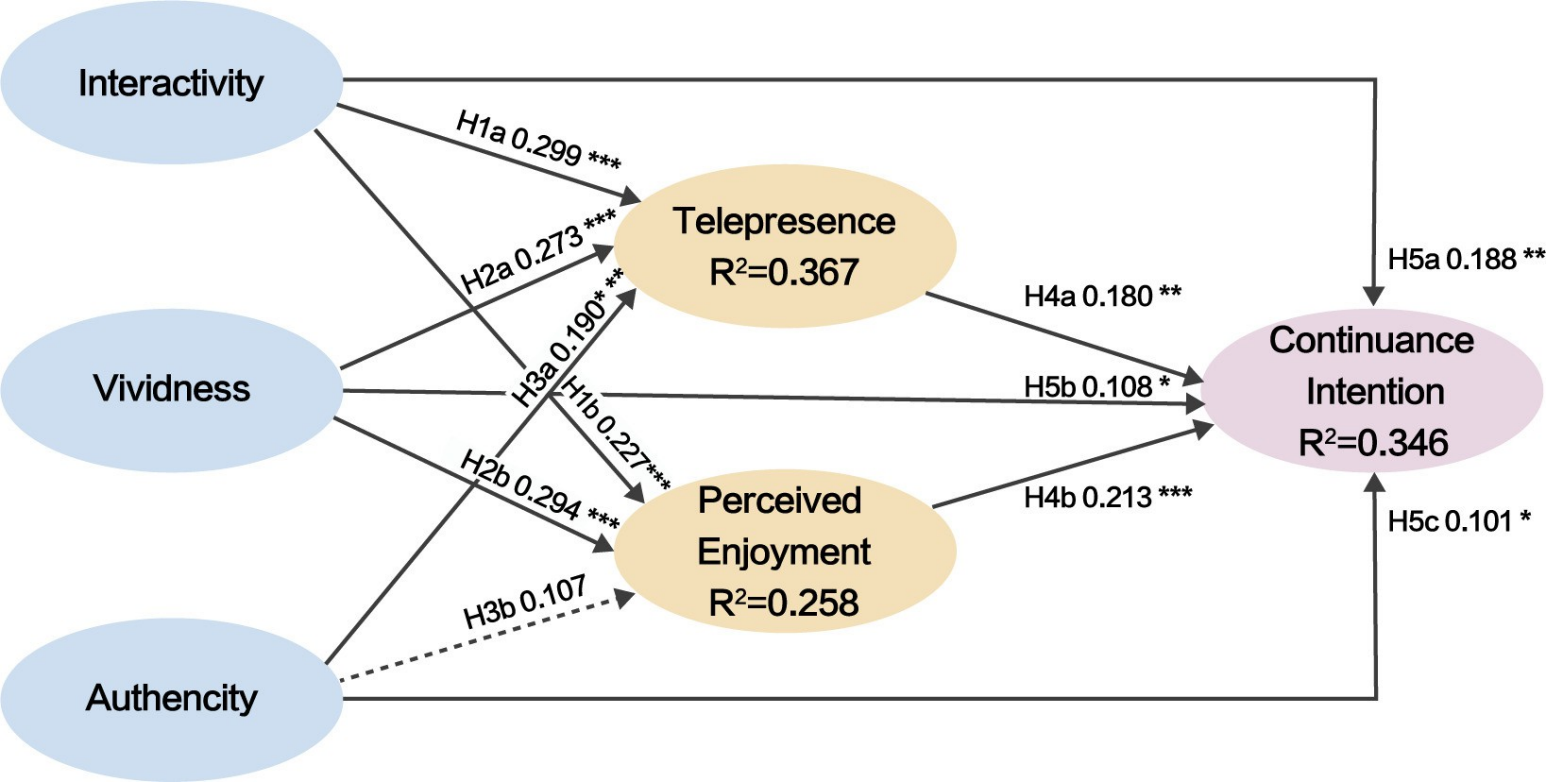
Model test result. (***p < 0.001, **p < 0.01, *p < 0.05, dot line: not significant).

Finally, to test model path significance, bootstrapping with 5,000 resamples was applied [127]. The standardized path coefficient (Std Beta) represents the change in the dependent variable for each standard deviation increase in an independent variable, with higher absolute values indicating stronger relationships. Hypotheses are generally considered statistically significant when the p-value is below 0.05. Additionally, confidence intervals help assess path coefficient significance: if the interval does not include zero, the path is significant at the 95% confidence level. Path analysis results are shown in Fig 4, with standardized coefficients in Tables 8 and 9. The results indicate that all hypotheses were supported except for H3b (AUT→PE) and H7c (AUT→PE→CI), where p-values exceeded 0.05 and confidence intervals included zero, indicating nonsignificant paths.

**Table 8:** Hypothesis test results.

Hypothesis	Path	Std Beta	*t*-value	*p*-value	2.5%	97.5%	Results
**H1a**	INT→TE	0.299	6.148	0.000	0.202	0.394	Supported
**H2a**	VIV→TE	0.273	5.882	0.000	0.179	0.359	Supported
**H3a**	AUT→TE	0.190	4.041	0.000	0.097	0.281	Supported
**H1b**	INT→PE	0.227	4.163	0.000	0.121	0.330	Supported
**H2b**	VIV→PE	0.294	5.767	0.000	0.194	0.395	Supported
**H3b**	AUT→PE	0.107	1.879	0.060	-0.004	0.219	Not Supported
**H4a**	TE→CI	0.180	3.184	0.001	0.069	0.291	Supported
**H4b**	PE→CI	0.213	4.675	0.000	0.125	0.303	Supported
**H5a**	INT→CI	0.188	3.324	0.001	0.076	0.297	Supported
**H5b**	VIV→CI	0.108	2.059	0.040	0.003	0.207	Supported
**H5c**	AUT→CI	0.101	2.166	0.030	0.012	0.195	Supported

*Note*. →represents the path relationship.

**Table 9:** Mediation hypothesis test results.

Hypothesis	Path	Std Beta	*t*-value	*p*-value	2.5%	97.5%	Results
**H6a**	INT→TE→CI	0.054	2.783	0.005	0.019	0.096	Supported
**H6b**	VIV→TE→CI	0.049	2.902	0.004	0.018	0.084	Supported
**H6c**	AUT→TE→CI	0.034	2.475	0.013	0.011	0.064	Supported
**H7a**	INT→PE→CI	0.048	3.022	0.003	0.020	0.083	Supported
**H7b**	VIV→PE→CI	0.063	2.902	0.004	0.033	0.099	Supported
**H7c**	AUT→PE→CI	0.023	1.688	0.092	-0.001	0.053	Not Supported
*Total effect*							
	INT→CI	0.102	4.036	0.000	0.057	0.156	
	VIV→CI	0.112	4.884	0.000	0.069	0.159	
	AUT→CI	0.057	2.838	0.005	0.021	0.098	

*Note*. →represents the path relationship.

## 5. Discussion

This study employs the S-O-R theory and telepresence to develop a user experience model for digital museums. It examines how interaction quality (vividness, interactivity, and authenticity) influences users’ continuance intention by stimulating psychological responses (telepresence and perceived enjoyment). The findings reveal key factors influencing the CDZM users’ continuance intention and highlight multiple mediating mechanisms.

### 5.1 Interaction quality and continuance intention

The analysis shows that perceived interaction quality (interactivity, vividness, and authenticity) significantly influences users’ continuance intention (supporting H5a, H5b, and H5c). Among these factors, interactivity exerts the greatest direct impact on continuance intention. This may be attributed to the functionality of the CDZM, which enables users to flexibly adjust perspectives, control content, access customized information, and utilize interactive help and search functions [45,94]. Such interactive experiences enhance users’ sense of immersion, engagement, and control [85,128–130], thereby triggering emotional responses that encourage continued platform use [131]. This finding is consistent with prior research in fields such as online travel and VR systems [93–95], emphasizing the critical role of interactivity in diversifying and personalizing user experiences.

In comparison, while vividness and authenticity exert a weaker influence on continuance intention than interactivity, their roles are still significant. Vividness enhances users’ immersion and enjoyment through multimedia presentations and rich sensory experiences, thereby strengthening their intention to continue using the system. This finding aligns with the multisensory stimulation theory [132] and previous empirical studies [24,96], indicating that multisensory stimuli facilitate both short-term engagement and long-term usage intentions. Authenticity also has a significant effect on users’ continuance intention. This result is consistent with prior research in virtual tourism and virtual reality gaming [133,134]. As a thematic cultural heritage museum, the CDZM attracts users seeking comprehensive, accurate, and authoritative cultural or historical information [135,136], such as detailed exhibits and precise reenactments of historical events. Consequently, users hold high expectations for authenticity. Meeting these expectations enhances their recognition of the museum’s educational and historical value, which, in turn, strengthens their intention to continue using its resources. Therefore, in virtual environments with user needs comparable to those of the CDZM, prioritizing investments in interactive features and vivid design elements, along with enhancing authenticity representation, is essential.

### 5.2 Interaction quality, telepresence, and perceived enjoyment

The interaction quality of digital museums (interactivity, vividness, and authenticity) influences users’ telepresence experience at varying levels, supporting H1a, H2a, and H3a. Specifically, interactivity has a significant positive impact on telepresence. This suggests that interactive features in the CZDM, such as clicking, rotating, and zooming exhibits, or participating in virtual tours, enable users to feel actively involved in exploring cultural or historical scenes, thus fostering an immersive experience. The underlying mechanism may stem from interactivity enhancing users’ perception of control over the virtual space, which enhances their sense of agency and engagement [44,45]. This transformation, from passive spectators to active participants [46], diverts users’ attention from the real world, directing their cognitive resources toward the virtual environment, enhancing their sense of immersion. Such findings align with prior discussions on the relationship between interactivity and user control [137] and are supported by empirical studies [138,139]. However, a study by Jang et al. indicates that interactivity’s effect on telepresence in virtual environments is not consistently positive, as insufficient interactivity diminishes its impact. This highlights the necessity for technological advancements in interactivity to further improve user experiences [140].

Similarly, vividness positively influences telepresence, consistent with previous research findings [45,71]. This suggests that digital museums, utilizing vibrant colors, high-quality images, videos, and audio effects, enhance users’ perception of virtual environments, creating the feeling of being physically present in an actual museum. This finding supports existing telepresence theories, which propose that highly interactive and vivid virtual environments effectively transport users from the physical world to the virtual one [46]. Additionally, the results indicate that authenticity exerts a significant impact on telepresence. High authenticity, demonstrated through realistic, complete, accurate, and trustworthy representations of museum exhibits [41], strengthens users’ trust and sense of realism, thereby deepening their immersive experiences [141]. For instance, the accuracy of artifacts, the historical context of exhibitions, and the meticulous reproduction of details enhance users’ immersion in virtual museums. This aligns with the CZDM’s core objective, namely to evoke cultural identification and emotional resonance in users by faithfully recreating historical and cultural heritage. Research by Hsu et al. supports this conclusion, showing that authenticity reinforces telepresence and helps users better understand the gap between their expectations and the reality of the experience [43]. When this gap is small, users are more likely to engage. Conversely, a lack of authenticity in exhibits disrupts users’ immersion and hinders the development of telepresence [142].

Interactivity and vividness have a positive effect on users’ perceived enjoyment, supporting H1b and H2b. This finding aligns with the research by Jung et al. on AR applications in cultural heritage tourism [143]. Of these factors, vividness has the strongest influence on perceived enjoyment. In digital museums, vividness is reflected in high-resolution images, clear audio, immersive audiovisual experiences, and interactive features such as virtual reality (VR) and 360-degree videos. These sensory stimuli activate pleasurable neural responses [144], significantly influencing users’ emotions and enhancing their enjoyment. Specifically, vivid visual and sensory information increases users’ cognitive engagement while fostering emotional involvement, thereby reinforcing their perception of enjoyment [52]. Interactivity also positively influences perceived enjoyment, potentially because high interactivity in virtual environments provides users with immediate feedback and a sense of autonomy. Instant feedback tailored to users’ needs and interests further amplifies their enjoyment. Additionally, allowing users to select content and modes based on their preferences increases satisfaction and overall enjoyment [78]. Another possible explanation is that interactivity improves system convenience, which has been shown to be associated with users’ enjoyment [145–147].

However, authenticity does not significantly influence perceived enjoyment (failing to support H3b), which contradicts our initial hypothesis. While existing literature indicates that authenticity typically enhances users’ enjoyment in virtual reality experiences [148], in our study, users’ need for authenticity may have been overshadowed by the stimulating effects of high interactivity and vividness. A highly authentic digital museum may offer knowledge-oriented and educational experiences, but without entertaining elements such as interactivity and gamified displays, users’ perceived enjoyment may remain low. One possible explanation is that perceived enjoyment is more contingent on factors associated with entertainment, such as sensory stimuli, interactivity, and immersion, than on the authenticity of the exhibits[149]. This finding highlights an important direction for future research: exploring how to balance authenticity with other quality attributes to enhance users’ enjoyment in various types of virtual experiences [74].

### 5.3 Telepresence, perceived enjoyment and continuance intention

Telepresence and perceived enjoyment significantly influence users’ continuance intention, supporting H4a and H4b. First, when users experience a strong sense of telepresence in a digital museum, they are more likely to perceive it as realistic and engaging, thereby increasing their intention to continue using the platform. Prior research also suggests that telepresence technologies enhance users’ immersive experiences with products or services, thereby fostering greater engagement [150]. Therefore, telepresence can be considered a critical factor influencing users’ continuance intention on virtual platforms [151,152]. This finding suggests that digital museums should prioritize enhancing virtual telepresence by leveraging high-quality virtual reality (VR) technologies to provide more immersive experiences, thus increasing user retention.

Second, higher levels of perceived enjoyment in digital museums significantly enhance users’ continuance intention [68,153] This may be because perceived enjoyment, as an intrinsic motivation, differs from extrinsic motivations (e.g., rewards or compensation) in its enduring nature and ability to inspire autonomous behavior [154]. This aligns with the principles of hedonic systems, which are characterized by excitement and drive enjoyment-related behaviors [155]. Consequently, if users derive enjoyment from participating in exhibitions, activities, or interactions in the digital museum, this intrinsic enjoyment will motivate revisits.

### 5.4 The mediating role of telepresence and perceived enjoyment

When exploring how interactive quality affects users’ continuance intention, the mediating role of telepresence and perceived enjoyment is revealed. First, telepresence serves as a significant mediator between interaction quality and continuance intention, supporting H6a, H6b, and H6c. Users who experience high interactivity, vividness, and authenticity in a digital museum are more likely to feel fully immersed. This telepresence enhances engagement and involvement, significantly boosting users’ continuance intention. Specifically, interactivity enhances telepresence by providing users with a sense of control and immediate feedback. Vividness deepens immersion through rich visual and auditory stimuli [45], while authenticity improves the credibility of the virtual environment. Together, these factors strengthen telepresence, ultimately encouraging users to remain engaged with digital museums [47]. The mediating pathway highlights the dual role of telepresence as both an emotional and cognitive driver, revealing its multifaceted contribution to strengthening the positive effect of interaction quality on continuance intention. This finding supports and extends telepresence theory [156]. Although prior studies have validated the direct effects of perceived interaction quality on telepresence and continuance intention [43,45,71,74,68,87], few have examined its mediating role. This study bridges this gap by confirming the mechanism within the context of digital museums, providing new insights for understanding how continuance intention forms in complex digital environments.

Second, perceived enjoyment mediates the relationship between interactivity, vividness, and continuance intention, supporting H7a and H7b, with a stronger mediating effect than telepresence. This suggests that perceived enjoyment is a key driver of users’ continuance intention. As an intrinsic motivator [157], perceived enjoyment not only provides immediate satisfaction but also fosters a long-term emotional connection to the platform [158]. This emotional bond significantly shapes users’ attitudes and behaviors. In contrast, the impact of telepresence on continuance intention is less direct and enduring than perceived enjoyment, as it lacks emotional drive. This mediating mechanism shows that high-quality interactivity and vividness directly enhance users’ enjoyment and indirectly influence their continuance intention via perceived enjoyment [96]. Specifically, interactive and vivid sensory experiences evoke enjoyment and satisfaction for users [42]. This perceived enjoyment serves as a primary motivator for their willingness to continue using the platform. These findings emphasize the importance of perceived enjoyment in designing and developing digital museums. However, perceived enjoyment does not significantly mediate the relationship between authenticity and continuance intention, failing to support H7c. This result may stem from authenticity primarily affects users’ trust and perceived content credibility rather than directly generating enjoyment. Therefore, while enhancing content authenticity, digital museums should also focus on other strategies, such as improving interactivity and vividness, to boost users’ enjoyment and achieve greater continuance intention.

## 6. Conclusions and suggestions

In today’s digital age, creating immersive and innovative museum experiences through digital technology has become a critical focus for cultural institutions, educators, and developers. This study integrates the S-O-R model with telepresence theory to develop a structural equation model for investigating users’ continuance intention in digital museums. It examines the mechanisms through which interaction quality (interactivity, vividness, and authenticity) and users’ psychological responses influence their continuance intention. The results demonstrate that interaction quality significantly impacts users’ continuance intention, with telepresence serving as a key mediator. Perceived enjoyment significantly mediates the effects of interactivity and vividness on continuance intention, but not that of authenticity. This study extends the S-O-R model and telepresence theory to the digital culture domain, enhancing insights into user psychology and behavioral motivations. It also provides empirical guidance for designing and operating digital museums, enabling practitioners to develop targeted strategies to enhance user experience and retention, and support digital cultural dissemination and educational advancement.

### 6.1 Theoretical implications

Theoretically, this study makes three main contributions:

Firstly, it introduces a novel model for understanding user experience in digital museums, addressing the limitations of traditional models such as the technology acceptance model (TAM) and the expectation confirmation model (ECM). While these models focus on direct relationships between factors such as system functionality, efficiency, and user satisfaction, they overlook the underlying psychological mechanisms. The new model explores the antecedents, mediators, and outcomes of user experience, with an emphasis on how digital technology influences psychological perception. Additionally, although previous research has explored the mediating role of perceived enjoyment between augmented reality and behavioral intention to use [159], it lacks depth. This article refines the understanding of telepresence and perceived enjoyment, showing their direct and indirect roles in interaction quality and continuance intention, and extends the application of telepresence and enjoyment theories in digital contexts.

Secondly, this study identifies the key elements of digital museum design. The research reveals that interactivity, vividness, and authenticity in digital museums play vital roles in directly or indirectly enhancing users’ continuance intention. By understanding these key factors, digital museum practitioners can effectively stimulate users’ positive emotions, meeting their needs for system design and service experience. Notably, authenticity is a relatively new factor in virtual experience research. While recent studies have focused more on activity-related authenticity [38,160], the role of constructive authenticity remains less explored. In fact, constructive authenticity is positively related to the sense of telepresence.

Finally, this study integrates and extends research on virtual experience. While previous research has emphasized the impact of telepresence and perceived enjoyment on overall user satisfaction and engagement, our research further refines these concepts by examining their roles in specific application scenarios. It investigates how the characteristics of interaction quality influence users’ continuance intention through telepresence and perceived enjoyment. This endeavor is crucial for understanding the role of virtual experience in specific applications. It offers novel insights for the study of virtual education, online retail, and other aspects of virtual environments and user experience, thereby enriching virtual experience research.

### 6.2 Practical implications

This study also provides practical guidance for designing and managing digital museums and other virtual environments, helping practitioners utilize the distinctive characteristics of these platforms to enhance the user experience.

#### 6.2.1 Enhancing the design and development of interaction quality

Firstly, a high level of interaction immerses users in the exhibition, providing a sense of telepresence and enjoyment. Therefore, various interactive features should be designed to enhance user engagement with digital museums. For example, 1) Develop a multi-level interactive interface that allows users to browse exhibits dynamically and explore them in a three-dimensional virtual space. Users can view exhibits from multiple angles and interact with them using virtual tools, increasing their control over the exhibits. 2) Enable real-time multi-user collaboration for simultaneous interaction, fostering exploration and discussion. 3) Encouraging users to create and share content, such as photos or comments related to exhibits, goes beyond interaction and represents higher participation, allowing users to shape their museum experience actively.

Secondly, the utilization of high-quality visual, auditory, and tactile technologies is recommended to enhance the impact of exhibits. Real-time rendering and high dynamic range imaging can create high-quality visual effects, while optimizing lighting and color in the virtual environment can provide different visual effects under varying light conditions. Additionally, the integration of high-quality audio elements, such as background music, interpretive audio, and ambient sound, can enhance the exhibition atmosphere and immersion. In particular, the application of 3D audio technology can provide a more realistic auditory experience for users.

Finally, the study reveals that digital museum users may prioritize interactivity and vividness over strict authenticity. However, authenticity remains crucial for enhancing users’ sense of telepresence. Museums can accurately reconstruct the historical background and cultural environment of exhibits through detailed historical research and cultural analysis to ensure content authenticity. This can include digital restoration of cultural relics, in-depth interpretation of background stories, and simulation of the exhibits’ environments. Additionally, the use of high-precision texture and material scanning technology allows for the accurate simulation of the surface details and textures, presenting a realistic appearance in the virtual environment.

#### 6.2.2 Considering the positive effect of users’ psychological feelings on their continuance intention

Firstly, focusing on users’ perceived enjoyment is crucial. In the highly competitive virtual experience market, it is a challenge for a digital museum to stand out and maintain user engagement. Therefore, optimizing perceived enjoyment is key to sustaining future competitiveness. Enhancing users’ enjoyment through increased vividness and interactivity is a primary driver of their continuance intention. Museums should create engaging content and interactive experiences, such as interactive stories, virtual tasks, or gaming elements, to encourage users to explore and learn. To maintain user engagement and interest, exhibition content should be regularly updated with new interactive elements, such as seasonal exhibitions, special events, and time-limited experiences.

Secondly, enhancing users’ telepresence is necessary. Telepresence refers to the deep sense of immersion users experience in a virtual environment. This feeling of “being there” is a key factor in driving continuance intention. By optimizing interactivity, vividness, and authenticity, museums can enhance users’ sense of telepresence. High-quality 3D environments, real-time interactions, fine texture processing, and accurate lighting effects all contribute to this sense of immersion.

These insights provide a deeper understanding of the interactive characteristics of digital museums, the role of telepresence, and the importance of perceived enjoyment in user experience. This provides a theoretical basis and practical guidance for designing and managing more engaging digital platforms, serving as valuable references for the future design of digital content platforms in education, culture, and business.

### 6.3 Policy implications

1. Establishing user experience standards and data-driven evaluation mechanisms. This study confirms the significant role of user experience factors in enhancing long-term user engagement with digital museums. These findings suggest that government and cultural institutions could improve the effective use of cultural resources and promote regional economic growth by implementing a standardized user experience evaluation system across the digital cultural industry. Accordingly, we recommend establishing user experience standards that include interaction quality, telepresence, and perceived enjoyment, along with a unified platform for data collection and analysis, to create a standardized evaluation system across the industry. Such a system would enable digital museums to leverage big data to precisely identify and respond to user needs and psychological responses, providing a scientific basis for policy-making and resource allocation. Furthermore, implementing transparent operational decisions and oversight systems in digital museums can strengthen public engagement and foster trust [161–163], ultimately enhancing user continuance intention.

2. Prioritizing support for interactive and immersive technologies. The findings indicate that interactive and immersive technologies (such as VR and AR) significantly impact users’ enjoyment and telepresence. To fully harness the potential of these technologies, economic policies should provide tax incentives, financial subsidies, and other support to encourage digital museums to adopt them. Such technologies can substantially improve digital museums’ performance in interaction quality, vividness, and authenticity, meeting users’ needs for enjoyment and telepresence and driving long-term engagement. These technologies also facilitate collaboration along related supply chains, fostering cultural consumption and broader use of cultural resources.

3. Promoting diversified industry training and cross-sector collaboration. Developing digital museum practitioners’ skills in data analysis and user behavior management directly impacts service quality and innovation. Therefore, policies should support diversified professional training in the industry, focusing on advancing skills in cutting-edge design concepts and data analysis to improve service quality and drive continuous innovation in user experience design. Additionally, policies should support cross-sector collaboration, enabling practitioners to gain insights from business management [164], crisis management [165,166], resource optimization [167], and user behavior management to address management and operational complexities more effectively. Furthermore, digital museums should be designated as core areas of support for cultural dissemination and education, with sustained financial and technological resources to fulfill their role in cultural promotion and public education [168], thereby extending the economic benefits of digital cultural services.

### 6.4 Limitations and future research directions

This study endeavors to maintain rigor and scientific integrity, and offers valuable insights. However, it is important to acknowledge its limitations, which are outlined below and should be considered in future research.

1. Variable scope. This study only analyzes three interactive qualities (interactivity, vividness, and authenticity) and two psychological perception dimensions (telepresence and perceived enjoyment) in the digital museum experience, which may not be comprehensive. Future research could introduce more variables, such as effectiveness and flow, to gain a more nuanced understanding of user experience across multiple dimensions.

2. Sample representation. The data for this study were collected through surveys of users interested in the CZDM, which could limit the generalizability of the results. Given the diversity of user groups, future studies should examine these findings across different age groups (e.g., older adults, children) and social roles (e.g., students, entrepreneurs) [169]. Additionally, validating the results in various application scenarios, such as virtual shopping or digital education, is recommended. Such efforts are expected to enhance the generalizability and practical relevance of the conclusions.

3. Methodological constraints. This study employed self-reported online questionnaires to collect data. While this method effectively captured users’ subjective experiences, it also has certain limitations. First, social desirability bias may have led respondents to align their answers with social expectations when evaluating their interaction experiences and continuance intentions, potentially overstating associations between variables. Additionally, although the questionnaires were administered immediately after users visited the digital museum, thereby minimizing the risks of traditional recall bias, instantaneous attention bias and transient emotional bias might still have occurred. Instantaneous attention bias refers to users being impressed by specific prominent designs or content, potentially leading to an incomplete evaluation of the overall experience. Transient emotional bias occurs when users’ responses are influenced by emotional fluctuations during the experience, possibly amplifying the perceived impact of specific aspects. Although these biases are relatively minor, they may constrain the model’s explanatory power. To mitigate these biases, future studies could incorporate more objective measurement methods, including system log data to quantify continuance intentions or behavioral tracking and physiological measures such as eye-tracking and facial expression analysis. Additionally, a triangulation approach, integrating qualitative interviews, experimental controls, and multi-source data, could strengthen the robustness and external validity of the findings.

4. Cultural differences. This study focuses on the CZDM, with Zisha culture being a significant element of traditional Chinese culture. However, its specific content may limit the applicability of the findings to digital museums outside the scope of Zisha culture. As the audience for Zisha culture is relatively niche, their psychological needs and interaction styles may differ from those in other themed museums. Therefore, the generalizability of this study’s findings should be cautiously interpreted. Future research could explore more varied museum types to further validate these conclusions. Additionally, users from diverse cultural backgrounds may exhibit different responses to telepresence and perceived enjoyment. Since this study primarily examines user experience within Chinese culture, future research should consider cross-cultural comparisons to gain a more comprehensive understanding of the global impact of these factors.

5. Gap between intentions and actions. This study primarily examines users’ continuous intentions, without tracking actual behaviors (e.g., physical museum visits or continuous digital engagement). Prior research indicates that intentions do not always accurately reflect actual behavior, potentially constraining our findings’ interpretability. Thus, we recommend caution when applying these results to predict real-world behaviors, and suggest that future research improve the model’s applicability by incorporating objective behavior-tracking methods. For instance, tracking users’ visit frequency and duration at physical museums, as well as their continuous engagement on digital platforms, could yield behavior data to validate the translation of intentions into actions. Additionally, collecting multi-wave data to monitor behavioral changes over time could offer a comprehensive view of user behavior while improving model accuracy and external validity.

## Supporting information

S1 Data (XLSX)
